# Microarray data analysis on gene and miRNA expression to identify biomarkers in non-small cell lung cancer

**DOI:** 10.1186/s12885-020-06829-x

**Published:** 2020-04-16

**Authors:** Xiang Jin, Yinghui Guan, Zhen Zhang, Hongyue Wang

**Affiliations:** 1 Department of Respiration, The First Hospital of Jilin University, No. 1 Xinminda Street, Changchun, 130021 China; 2 PICU, The First Hospital of Jilin University, Changchun, 130021 China; 3 Department of Nephrology, The First Hospital of Jilin University, Changchun, 130021 China

**Keywords:** Non-small cell lung cancer, Differentially expressed genes, miRNA, Regulatory network, Microarray data analysis

## Abstract

**Background:**

The aim of this study was to gain further investigation of non-small cell lung cancer (NSCLC) tumorigenesis and identify biomarkers for clinical management of patients through comprehensive bioinformatics analysis.

**Methods:**

miRNA and mRNA microarray datasets were downloaded from GEO (Gene Expression Omnibus) database under the accession number GSE102286 and GSE101929, respectively. Genes and miRNAs with differential expression were identified in NSCLC samples compared with controls, respectively. The interaction between differentially expressed genes (DEGs) and differentially expressed miRNAs (DEmiRs) was predicted, followed by functional enrichment analysis, and construction of miRNA-gene regulatory network, protein-protein interaction (PPI) network, and competing endogenous RNA (ceRNA) network. Through comprehensive bioinformatics analysis, we anticipate to find novel therapeutic targets and biomarkers for NSCLC.

**Results:**

A total of 123 DEmiRs (5 up- and 118 down-regulated miRNAs) and 924 DEGs (309 up- and 615 down-regulated genes) were identified. These genes and miRNAs were significantly involved in different pathways including adherens junction, relaxin signaling pathway, and axon guidance. Furthermore, hsa-miR-9-5p, has-miR-196a-5p and hsa-miR-31-5p, as well as hsa-miR-1, hsa-miR-218-5p and hsa-miR-135a-5p were shown to have higher degree in the miRNA-gene regulatory network and ceRNA network, respectively. Furthermore, *BIRC5* and *FGF2*, as well as *RTKN2* and *SLIT3* were hubs in the PPI network and ceRNA network, respectively.

**Conclusion:**

Several pathways (adherens junction, relaxin signaling pathway, and axon guidance) miRNAs (hsa-miR-9-5p, has-miR-196a-5p, hsa-miR-31-5p, hsa-miR-1, hsa-miR-218-5p and hsa-miR-135a-5p) and genes (*BIRC5*, *FGF2*, *RTKN2* and *SLIT3*) may play important roles in the pathogenesis of NSCLC.

## Background

Lung cancer presents the major cause of cancer deaths in the past few decades and has been a major public health problem [1]. Histologically, non-small cell lung cancer (NSCLC) accounts for more than 80% of lung malignancy cases, with an overall 5-year survival rate below 15% [2]. More than 70% of these cases are diagnosed with locally advanced or metastatic disease and the prognosis for NSCLC patients remains poor [3]. Therefore, it places a high priority on elucidating the molecular mechanisms of NSCLC pathogenesis and identify diagnostic or predictive biomarkers.

Research has revealed significant interactions between gene alterations and tumorigenesis and tumor progression of many types of cancers [4]. CD44 was shown to be overexpressed in NSCLC and involved in the occurrence and migration of NSCLC [5]. The genes *L1TD1* (LINE-1 Type Transposase Domain Containing 1) and *SPAG6* (Sperm Associated Antigen 6) are tumor-specifically methylated in NSCLC [6]. Recently, Morris et al. reported that *FPR1* mRNA levels in whole blood predicts both NSCLC and small cell lung cancer [7]. MicroRNAs (miRNAs) are a large group of small non-coding RNAs of 20–24 nucleotides that are involved in the fine-tuning of various biological processes. They bind to multiple target mRNAs typically in the 3′-untranslated region (3′-UTR) and govern gene expression at the post-transcriptional level [8]. A possible tumor suppressor role for hsa-miR-30d in progression of NSCLC was shown in a recent study [9]. Yang et al. demonstrated that miR-598 suppressed the invasion and migration in NSCLC as a tumor suppressor through negatively regulate Derlin-1 (DERL1) and epithelial-mesenchymal transition (EMT) [10]. However, the roles of genes and miRNAs in NSCLCs are still not well understood [11].

Expression profiling with high-throughput microarrays has become a mature and widely used technology to obtain more global views on cancer genes and to identify novel cancer biomarkers [12]. In this study, we reported an integrated analyses of miRNAs and gene expression by reanalyzing public datasets from GSE102286 and GSE101929. Differentially expressed genes (DEGs) and differentially expressed miRNAs (DEmiRs) were identified in NSCLC samples compared with controls, respectively. The interaction between DEmiRs and DEGs was predicted, followed by functional enrichment analysis, and construction of miRNA-gene regulatory network, protein-protein interaction (PPI) network, and competing endogenous RNA (ceRNA) network. Through comprehensive bioinformatics analysis, we anticipate to find novel therapeutic targets and biomarkers for NSCLC.

## Methods

### Source of the microarray data

The public Gene Expression Omnibus (GEO) repository at http://www.ncbi.nlm.nih.gov/geo/ serve as a public data archive that freely disseminates high-throughput functional genomic data [13]. In this study, miRNA and mRNA microarray datasets were downloaded from the GEO database under the accession number GSE102286 and GSE101929, respectively. The miRNA dataset GSE102286 included 88 normal control samples and 91 NSCLC samples. All samples were tested using the GPL23871 NanoStringnCounter Human miRNA Expression Assay v1.6 platform. The gene expression profile GSE101929 included 34 normal control samples and 32 NSCLC samples. The platform used for the gene microarray was GPL570 [HG-U133_Plus_2] Affymetrix Human Genome U133 Plus 2.0 Array.

Patients that underwent curative NSCLC surgery between 1998 and 2014 were enrolled. The mRNA and miRNA cohorts included matched tumor and normal pairs. The mRNA cohort included 25 matched pairs and 7 unmatched pairs and the miRNA cohort included 93 matched pairs and two unmatched pairs.

### Data preprocessing and DEmiRs and DEGs screening

The miRNA raw data (RCC files) were received and read into the R statistical environment, followed by data preprocessing using the NanoStringNorm package [14] (v1.2.1, https://cran.r-project.org/web/packages/NanoStringNorm/), including background correction, normalization, and concentration prediction. The results of non-human miRNA probes on the chip were removed, and the remnant was used as the final miRNA expression value.

In addition, the mRNA raw data (CEL files) and the platform annotation file were downloaded. The affy package [2] was used for data preprocessing, including background correction, normalization, and expression level calculation. In accordance with the platform annotation information, the probes were converted into gene symbols. For multiple probes corresponding to the same gene symbol, the probe average was taken as the final expression value of gene.

The expression matrix was divided into tumor group and normal group, and DEmiRs and DEGs were screened separately. Non-paired t-test provided by limma [15] were used to calculate the *P*-values of significant gene expression differences. Benjamini–Hochberg (BH)-corrected *P*-values < 0.05 and |log_2_fold change (FC)| > 1 were chosen as the threshold for the identification of significant DEmiRs and DEGs.

### Identification of DEmiR-DEGs relationship pairs

miRWalk 2.0 tools [16] was applied to predict the target genes of the top 10 up- and down-regulated miRNAs with high FC value. The miRNA-target gene regulatory data from at least 5 of the 7commonly used databases (miRWalk, miRanda, miRDB, miRMap, miRNAMap, RNA22, Targetscan) were collected. The obtained miRNA-target relationship was matched with DEGs obtained by microarray analysis to obtain the interaction between DEmiRs and DEGs (DEmiR-DEGs relationship pairs).

### Pathway enrichment analysis of miRNAs and genes

The Kyoto Encyclopedia of Genes and Genomes (KEGG) [17] function enrichment analysis of miRNAs involved in the DEmiR-DEG interaction was carried out using R clusterprofiler package [18], and the pathways with *P*-value < 0.05 and count ≥2 were significantly enriched.

KEGG [17] pathway enrichment analysis was also conducted on the up-and down-regulated genes involved in the DEmiR-DEG relationship pairs. The commonly used DAVID [7] (version6.8, https://david-d.ncifcrf.gov/) was applied for the function enrichment analysis with gene count≥2 and *P*-value < 0.05 as the thresholds.

### Construction of miRNA-gene regulatory network and module analysis

Based on the interaction information of the DEmiR-DEG, the construction of miRNA-gene regulatory network was performed using the Cytoscape software (vesrion: 3.2.0) [19]. The topological properties of network nodes were analyzed, such as the centrality of nodes.

### PPI network of the DEGs involved in DEMIR-DEG

The STRING database [20] (version: 10.0) was used to predict the PPI pairs of proteins encoded by DEGs involved in the DEmiR-DEG. The input gene set was DEGs and the species was *Homo sapiens*. The parameter of PPI score was set as 0.4 (indicating medium confidence) [21]. A PPI network involved in DEmiR-DEG was constructed by Cytoscape software (version: 3.2.0) [19].

### Construction of ceRNA network

ceRNA can act as decoys for miRNA binding and form complex regulatory networks based on an “miRNA response element (MRE) language” that regulate the abundance of any MRE-containing RNA transcripts such as long noncoding RNAs (lncRNAs) [22]. We used the miRNA-lncRNA interaction pairs in the starBase database (http://starbase.sysu.edu.cn/) [23] to screen the lncRNAs that have interaction with the DEMIR. The screening criteria for screening miRNA-lncRNA regulatory relationship pairs was medium stringency ≥2 and number of cancer types ≥1. Baes on the predicted miRNA-lncRNA relationship pairs and DEmiR-DEG (miRNA-mRNA) regulatory relationships obtained above, the lncRNA-miRNA-mRNA network (ceRNA network) was constructed using Cytoscape software.

## Results

### Identification of DEGs and DEmiRs

**Fig. 1:**
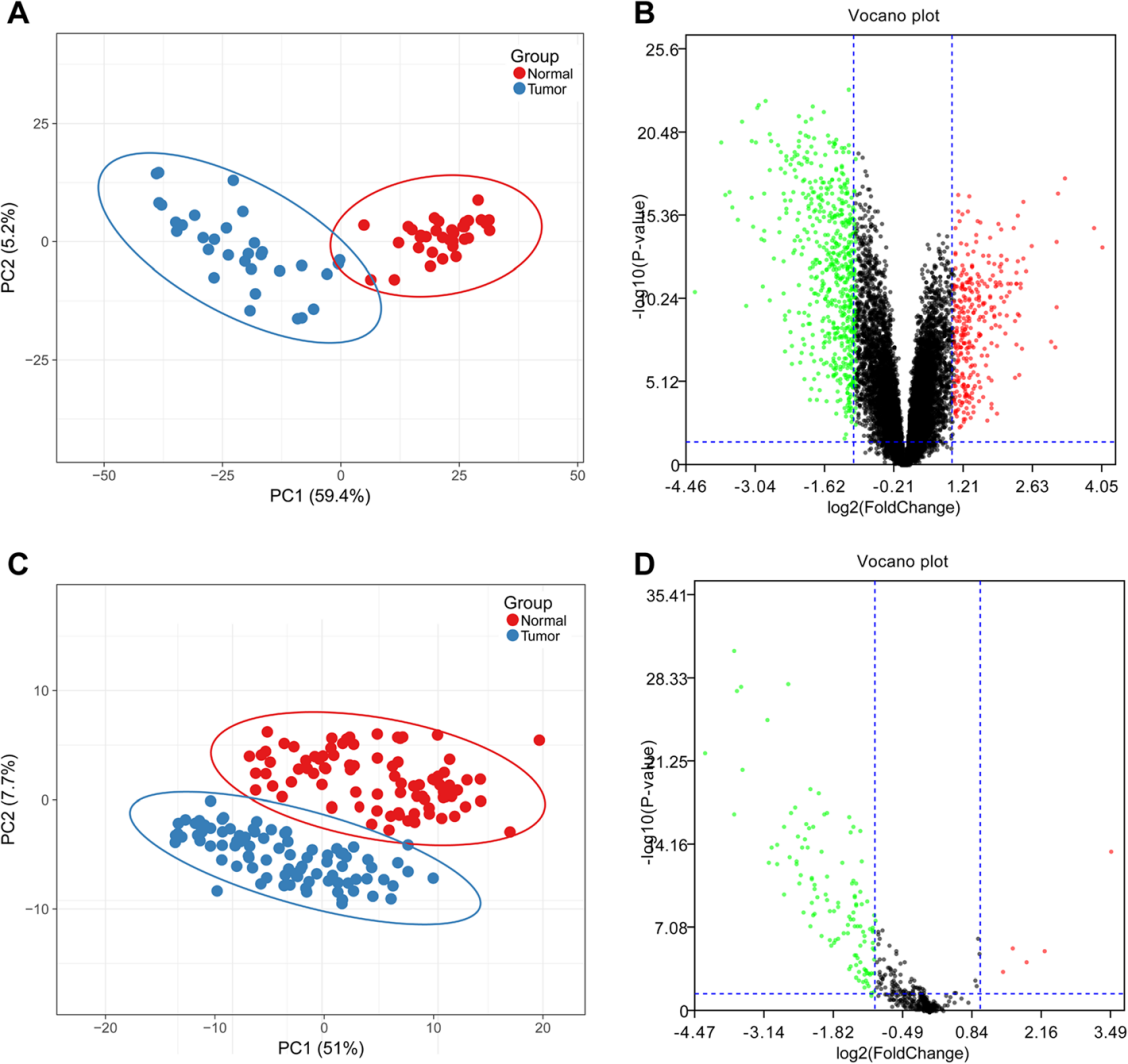
Principal component analysis (PCA) and volcano plots of differentially expressed genes (DEGs) and differentially expressed miRNAs (DEmiR). **a**, PCA of DEGs. **b**, volcano plot of DEGs. **c**, PCA of DEmiR. **d**, volcano plot of DEmiR

After data preprocessing, a total of 924 DEGs were obtained, of which 309 genes were significantly up-regulated and 615 were significantly down-regulated. The principal component analysis (PCA) and volcano plots of DEGs were shown in Fig. 1a and b. Additionally, 123 DEmiRs were identified, among which 5 miRNAs were significantly up-regulated and 118 miRNAs were significantly down-regulated. PCA and volcano plots of those DEmiRs were shown in Fig. 1c and d.

Then, by searching the miRWalk2.0 database, we found target genes for 18 mature miRNAs. Finally, we obtained 715 pairs of DEmiR-DEG, including 6 up- and 12 down-regulated miRNAs, as well as 101 up- and 245 down-regulated genes.

### Functional enrichment analysis of DEmiRs and DEGs

**Fig. 2:**
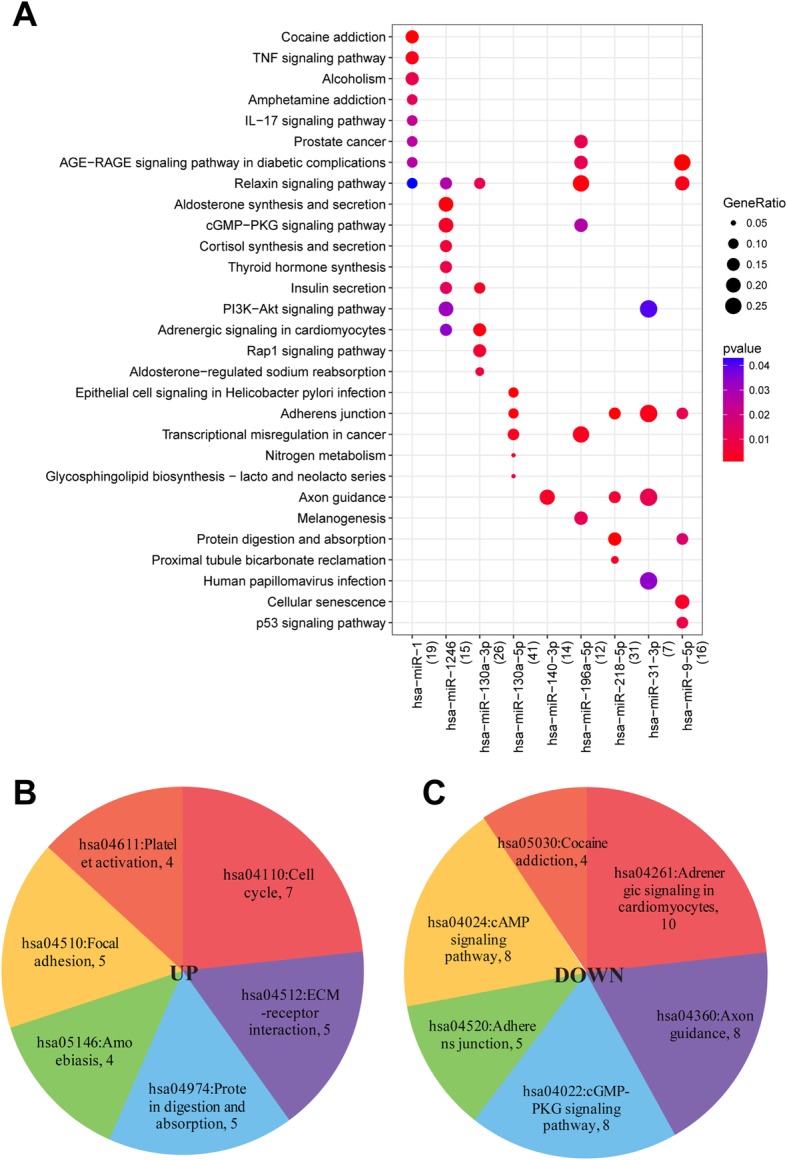
Kyoto Encyclopedia of Genes and Genomes (KEGG) pathways enriched by miRNAs or genes involved in DEmiR-DEGs. **a**, KEGG pathways enriched by 8 miRNAs of DEmiR-DEGs. **b**, KEGG pathway enriched by the up-regulated genes. **c**, KEGG pathway enriched by down-regulated genes

Totally, 66 KEGG pathways were enriched by 8 miRNAs of DEmiR-DEG (Fig. 2a and Table 1). Among these, adherens junction, relaxin signaling pathway, axon guidance, and transcriptional misregulation in cancer were significantly enriched by several miRNAs, such as hsa-miR-9-5p, hsa-miR-31-3p, hsa-miR-218-5p and hsa-miR-196a-5p. In addition, KEGG pathways enriched by up- and down-regulated genes (Fig. 2b and c) showed that up-regulated genes were significantly enriched in 6 KEGG pathways (cell cycle, protein digestion and absorption, ECM-receptor interaction, focal adhesion, amoebiasis, and Platelet activation) and down-regulated genes were significantly enriched in 6 KEGG pathways (adrenergic signaling in cardiomyocytes, axon guidance, cGMP-PKG signaling pathway, adherens junction, cAMP signaling pathway, and cocaine addiction).

**Table 1:** The enriched pathways by differentially expressed miRNAs

miRNA	ID	Pathway	*P* value	Count
hsa-miR-9-5p	hsa04933	AGE-RAGE signaling pathway in diabetic complications	4.84E-05	4
	hsa04926	Relaxin signaling pathway	2.51E-03	3
	hsa04218	Cellular senescence	4.51E-03	3
	hsa04115	p53 signaling pathway	9.18E-03	2
	hsa04520	Adherens junction	1.03E-02	2
	hsa04350	TGF-beta signaling pathway	1.38E-02	2
	hsa04974	Protein digestion and absorption	1.57E-02	2
	hsa05146	Amoebiasis	1.77E-02	2
	hsa05142	Chagas disease (American trypanosomiasis)	1.99E-02	2
hsa-miR-31-3p	hsa04520	Adherens junction	1.90E-03	2
	hsa04360	Axon guidance	1.08E-02	2
	hsa05165	Human papillomavirus infection	3.37E-02	2
	hsa04151	PI3K-Akt signaling pathway	4.04E-02	2
hsa-miR-218-5p	hsa04974	Protein digestion and absorption	3.14E-05	5
	hsa04520	Adherens junction	2.12E-04	4
	hsa04964	Proximal tubule bicarbonate reclamation	4.06E-03	2
	hsa04360	Axon guidance	5.77E-03	4
hsa-miR-196a-5p	hsa04926	Relaxin signaling pathway	1.04E-03	3
	hsa05202	Transcriptional misregulation in cancer	2.90E-03	3
	hsa05215	Prostate cancer	1.03E-02	2
	hsa04933	AGE-RAGE signaling pathway in diabetic complications	1.07E-02	2
	hsa04916	Melanogenesis	1.11E-02	2
	hsa04022	cGMP-PKG signaling pathway	2.75E-02	2
	hsa04020	Calcium signaling pathway	3.37E-02	2
hsa-miR-140-3p	hsa04360	Axon guidance	3.90E-03	3
hsa-miR-130a-5p	hsa05120	Epithelial cell signaling in Helicobacter pylori infection	5.11E-04	4
	hsa04520	Adherens junction	6.35E-04	4
	hsa05202	Transcriptional misregulation in cancer	3.41E-03	5
	hsa00910	Nitrogen metabolism	3.86E-03	2
	hsa00601	Glycosphingolipid biosynthesis - lacto and neolacto series	9.62E-03	2
	hsa04666	Fc gamma R-mediated phagocytosis	1.37E-02	3
hsa-miR-130a-3p	hsa04261	Adrenergic signaling in cardiomyocytes	1.47E-03	4
	hsa04911	Insulin secretion	3.14E-03	3
	hsa04015	Rap1 signaling pathway	5.38E-03	4
	hsa04960	Aldosterone-regulated sodium reabsorption	7.33E-03	2
	hsa04926	Relaxin signaling pathway	1.02E-02	3
	hsa04961	Endocrine and other factor-regulated calcium reabsorption	1.16E-02	2
hsa-miR-1246	hsa04925	Aldosterone synthesis and secretion	8.59E-04	3
	hsa04022	cGMP-PKG signaling pathway	3.92E-03	3
	hsa04927	Cortisol synthesis and secretion	6.97E-03	2
	hsa04918	Thyroid hormone synthesis	9.52E-03	2
	hsa04911	Insulin secretion	1.24E-02	2
	hsa04922	Glucagon signaling pathway	1.79E-02	2
	hsa04928	Parathyroid hormone synthesis, secretion and action	1.89E-02	2
	hsa04725	Cholinergic synapse	2.09E-02	2
	hsa04724	Glutamatergic synapse	2.17E-02	2
	hsa04926	Relaxin signaling pathway	2.77E-02	2
	hsa04728	Dopaminergic synapse	2.81E-02	2
	hsa04915	Estrogen signaling pathway	3.05E-02	2
	hsa04151	PI3K-Akt signaling pathway	3.17E-02	3
	hsa04261	Adrenergic signaling in cardiomyocytes	3.34E-02	2
	hsa04310	Wnt signaling pathway	3.43E-02	2
	hsa04934	Cushing’s syndrome	3.78E-02	2
hsa-miR-1	hsa05030	Cocaine addiction	2.45E-04	3
	hsa04668	TNF signaling pathway	2.47E-03	3
	hsa05034	Alcoholism	1.03E-02	3
	hsa05031	Amphetamine addiction	1.29E-02	2
	hsa04657	IL-17 signaling pathway	2.32E-02	2
	hsa05215	Prostate cancer	2.51E-02	2
	hsa04933	AGE-RAGE signaling pathway in diabetic complications	2.61E-02	2
	hsa04922	Glucagon signaling pathway	2.81E-02	2
	hsa04060	Cytokine-cytokine receptor interaction	3.02E-02	3
	hsa04152	AMPK signaling pathway	3.78E-02	2
	hsa04926	Relaxin signaling pathway	4.31E-02	2
	hsa04550	Signaling pathways regulating pluripotency of stem cells	4.86E-02	2
	hsa05418	Fluid shear stress and atherosclerosis	4.86E-02	2

### MiRNA-gene regulatory network construction

**Fig. 3:**
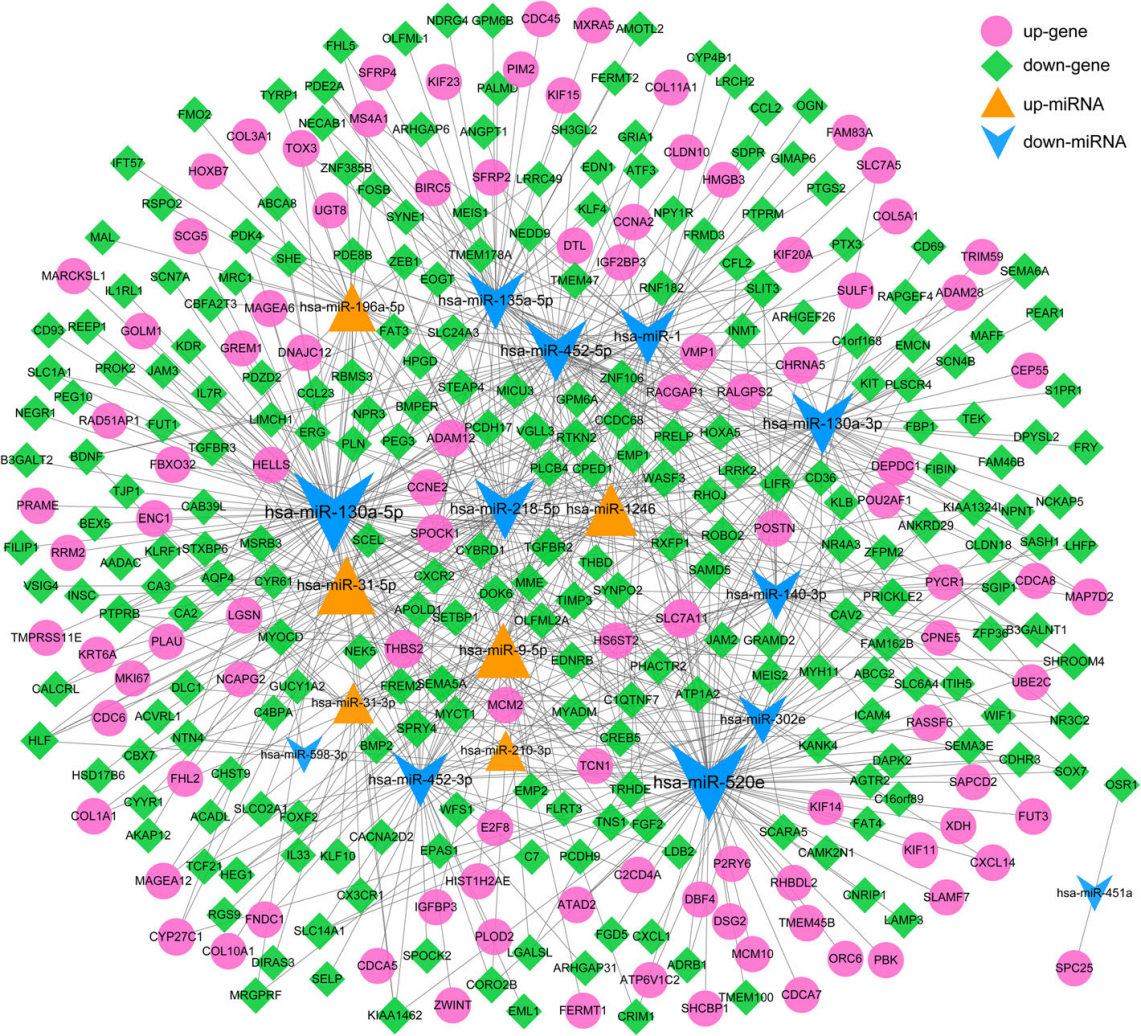
DEmiR-DEG regulatory network. Yellow trianglesrepresent up-regulated miRNAs, blue arrow indicates down-regulated miRNA, red circles represent up-regulated genes, and green prisms represent down-regulated genes

The DEmiR-DEG regulation network was shown in Fig. 3, consisting of 364 nodes and 715 interactions. The nodes with high topological score can be regarded as key network nodes. The top 5 miRNAs with higher degree included hsa-miR-130a-5p (down-regulated, degree = 93), hsa-miR-520e (down-regulated, degree = 90), hsa-miR-452-5p (down-regulated, degree = 56), hsa-miR-130a-3p **(**down-regulated, degree = 55), hsa-miR-218-5p **(**down-regulated, degree = 53). The top 5 genes with higher degree included Transforming Growth Factor Beta Receptor 2 (*TGFBR2*, down-regulated, degree = 8), Rhotekin 2 (*RTKN2*, down-regulated, degree = 7), Solute Carrier Family 7 Member 11 (*SLC7A11*, up-regulated, degree = 7), Slit Guidance Ligand 3 (*SLIT3*, down-regulated, degree = 7), and Semaphorin 5A (*SEMA5A*, down-regulated, degree = 7).

### Construction of PPI network

**Fig. 4:**
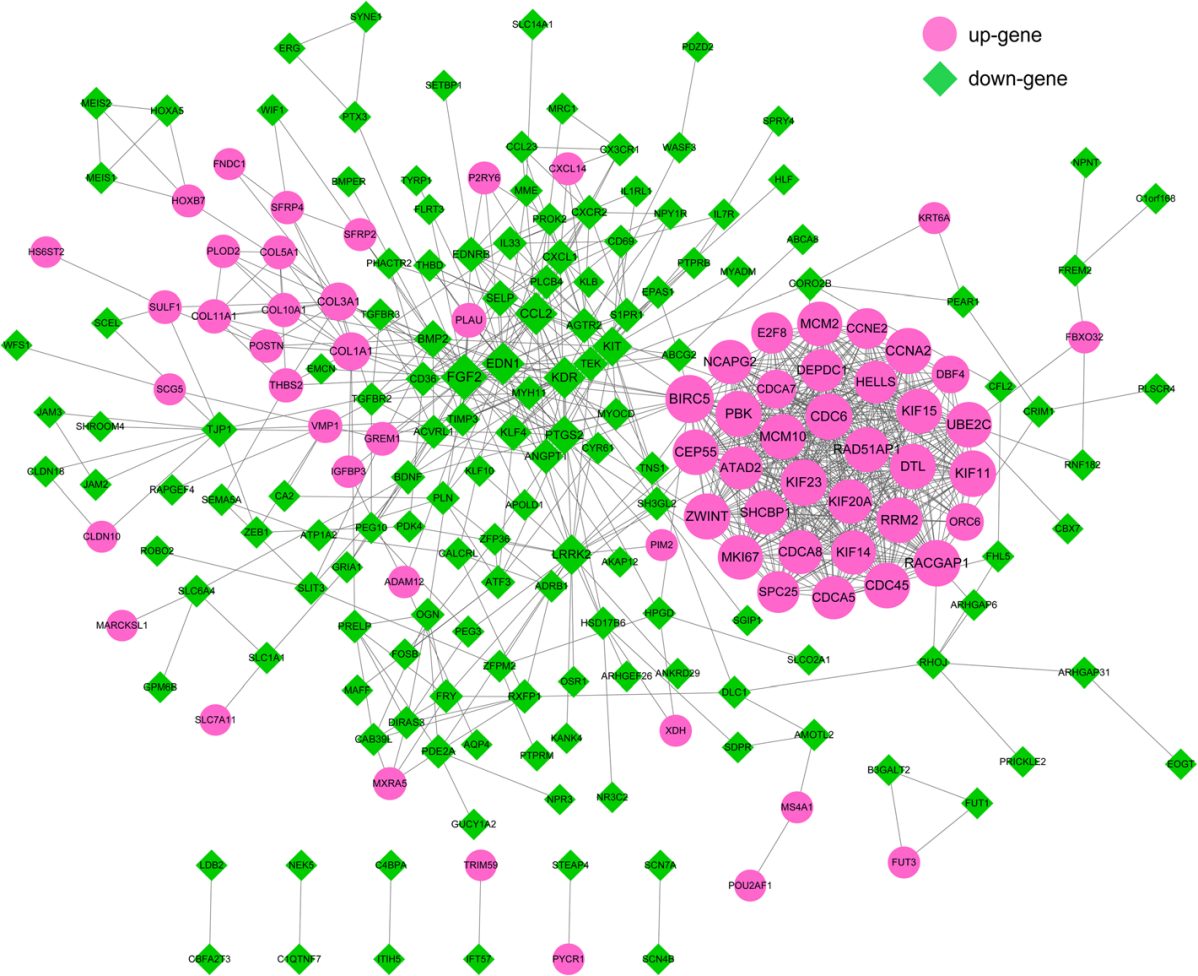
Protein-protein interaction network. The red circle indicates the up-regulated gene, the green prism represents the down-regulated gene, and the node size represents the degree value. The greater the degree value, the larger the node

The PPI network of genes involved in the DEmiR-DEG interaction was constructed as Fig. 4, including 860 interactions and 215 nodes (68 up- and 147 down-regulated genes). The top 10 genes with higher degree were as follows: Baculoviral IAP Repeat Containing 5 (*BIRC5*, up-regulated, degree = 36), Fibroblast Growth Factor 2 (*FGF2*, down-regulated, degree = 34), Rac GTPase Activating Protein 1 (*RACGAP1*, down-regulated, degree = 33), Cell Division Cycle 6 (*CDC6*, up-regulated, degree = 33), PDZ Binding Kinase (*PBK*, up-regulated, degree = 32), ZW10 Interacting Kinetochore Protein (*ZWINT*, up-regulated, degree = 32), Kinesin Family Member 11 (*KIF11*, up-regulated, degree = 32), Kinesin Family Member 23 (*KIF23*, up-regulated, degree = 32), Cyclin A2, (*CCNA2*, up-regulated, degree = 32), and Ribonucleotide Reductase Regulatory Subunit M2 (*RRM2*, up-regulated, degree = 32).

### ceRNA network construction

**Fig. 5:**
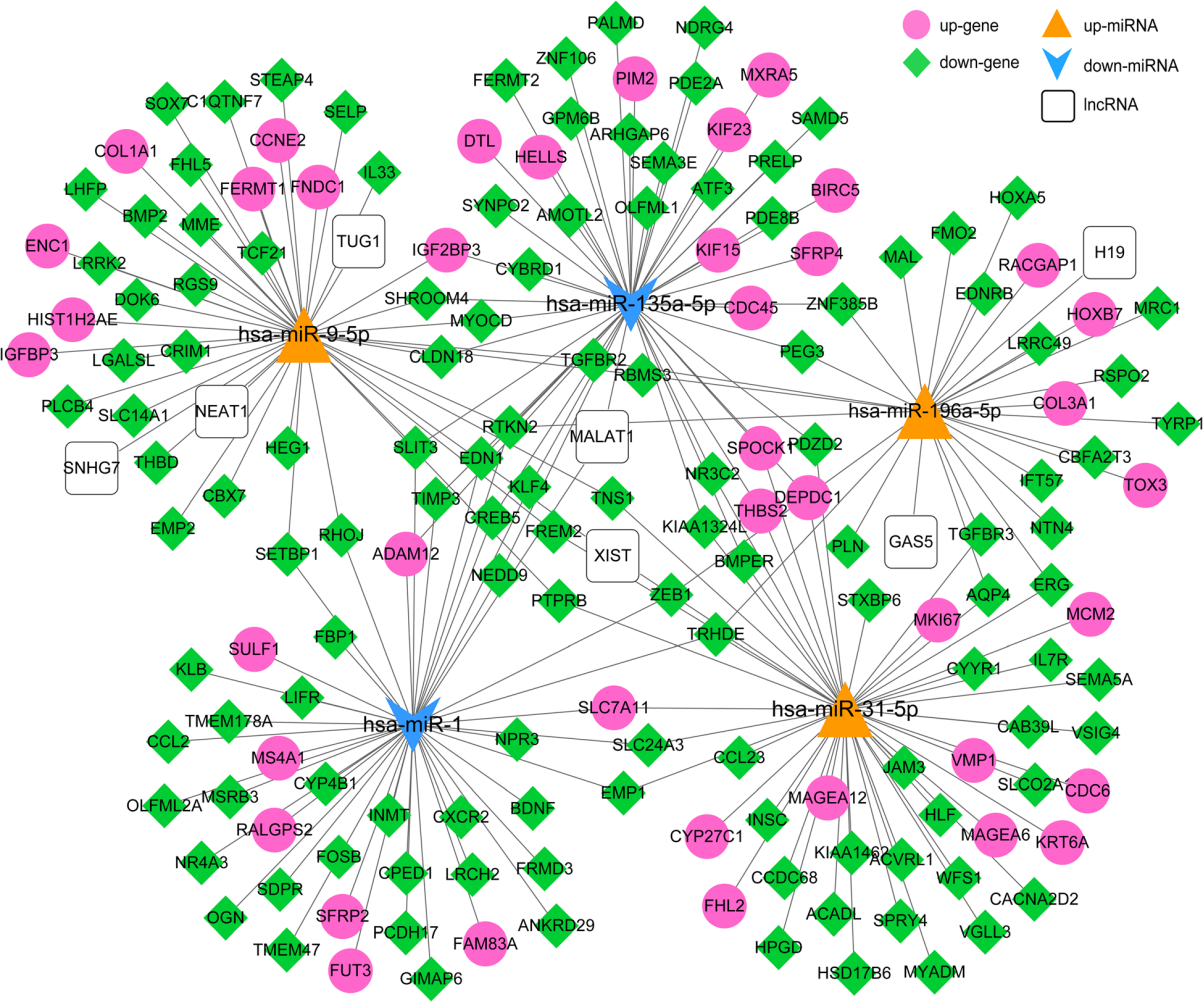
Competing endogenous RNA (ceRNA) network. The red circle represents the up-regulated gene, green prism represents the down-regulated gene, yellow triangle represents the up-regulated miRNA, blue arrow represents the down-regulated miRNA, and the white square is the lncRNA

The lncRNAs were predicted for all 18 miRNAs in the regulatory network, resulting in 9 miRNA-lncRNA relationships, including 5 miRNAs and 7 lncRNAs. We compared these 5 miRNAs in the regulation relationship of DEmiR-DEGs and integrated with miRNA-lncRNAs interaction. A total of 214 relationships were obtained, including 3 up-regulated miRNAs (hsa-miR-9-5p, has-miR-196a-5p, and hsa-miR-31-5p), 2 down-regulated miRNAs (hsa-miR-135a-5p and hsa-miR-1), 41 up-regulated genes, 124 down-regulated miRNAs, and 7 lncRNAs. The ceRNA network was shown in Fig. 5. The genes *RTKN2* and *SLIT3* had relative higher degree of 4 and 3, respectively. Both of *RTKN2* and *SLIT3* can be regulated by hsa-miR-1, hsa-miR-9-5p, and hsa-miR-135a-5p.

## Discussion

In this study, we integrated mRNA expression profile and miRNA expression profile to evaluate changes of gene and miRNA expression in NSCLC. A total of 123 DEmiRs (5 up- and 118 down-regulated miRNAs) and 924 DEGs (309 up- and 615 down-regulated genes) were found. These genes and miRNAs were significantly involved in different pathways including relaxin signaling pathway, adherens junction, and axon guidance. Hsa-miR-9-5p, has-miR-196a-5p and hsa-miR-31-5p, as well as hsa-miR-1, hsa-miR-218-5p and hsa-miR-135a-5p were shown to have higher degree in the miRNA-gene regulatory network and ceRNA network, respectively. Furthermore, *BIRC5* and *FGF2, as well as RTKN2* and *SLIT3*were hubs in the PPI network and ceRNA network, respectively.

Evidence suggests that relaxin, a peptide hormone, can promote invasiveness of breast cancer cell lines through increasing the expression of matrix metalloproteinases (MMPs) [24, 25]. A study has shown that mouse relaxin gene expression in the lungs can result in enhanced MMP activity [26]. However, there are few direct evidence targeting the role of relaxin in the progression of lung cancer. In this study, we found that the DEGs and DEmiR were associated with relaxin signaling pathway. Disruption of adherens junction has been demonstrated to play a causal role in cancer initiation and progression [27]. In addition, Notch1 destabilizates the adherens junction complex and influence NSCLC cell proliferation [28]. Cancer cells secrete axon guidance molecules control the invasion and migration of cancer [29]. In this study, we found that DEGs and DEmiRs were also involved in the adherens junction and axon guidance. Taken together, we suggested that relaxin signaling pathway, adherens junction, and axon guidance may play important roles in the pathogenesis of NSCLC.

In addition, we found several miRNAs had higher degrees in the constructed network, including hsa-miR-9-5p, has-miR-196a-5p, hsa-miR-31-5p, hsa-miR-1, hsa-miR-218-5p, and hsa-miR-135a-5p. Xu et al. reported up-regulation of miR-9 as poor prognostic biomarker in NSCLC patients [30]. A study showed that miR-196a could promote cell proliferation an invasion of NSCLC through regulating HOXA5 [31]. miR-31 acts as an oncogenic miRNA in lung cancer [32]. Moreover, there are evidence between the interaction of miR-1, miR-218, and miR-135a and lung cancer [33–35], which are in line with our study. Thus, we suggest the significant roles of hsa-miR-9-5p, has-miR-196a-5p, hsa-miR-31-5p, hsa-miR-1, hsa-miR-218-5p, and hsa-miR-135a-5p in NSCLC progression, which may be effective biomarkers used for the cancer diagnosis and therapy.

Furthermore, *BIRC5* and *FGF2*, as well as *RTKN2* and *SLIT3* were hubs in the PPI network and ceRNA network. Previous study showed that *BIRC5* was up-regulated in NSCLC [36]. FGF2-mediated autocrine signaling is activated in NSCLC cell lines [37]. Epigenetic inactivation of *SLIT3* has been found in human cancers [38]. However, there is no evidence regarding to the role of *RTKN2* in lung cancer. Nevertheless, we suggested that *BIRC5*, *FGF2*, *RTKN2* and *SLIT3* may be important for lung cancer; but experiment verification should be performed in future.

## Conclusion

In conclusion, the present study has demonstrated the potentially critical roles of several pathways (adherens junction, relaxin signaling pathway and axon guidance) miRNAs (hsa-miR-9-5p, has-miR-196a-5p, hsa-miR-31-5p, hsa-miR-1, hsa-miR-218-5p, and hsa-miR-135a-5p) and genes (*BIRC5*, *FGF2*, *RTKN2* and *SLIT3*) in the progression of NSCLC. The miRNAs and genes may be favorable biomarkers for patients with this cancer. However, there were still some limitations. Firstly, not all mRNA samples were from miRNA samples, which will reduce the statistical power for examining the relationship between miRNA and mRNA. Secondly, this findings were identified in one cohort, which should be confirmed in other cohorts. And hence future validations with the miRNA and mRNA samples from the same patients should be conducted.

## Data Availability

In this study, miRNA and mRNA microarray datasets were downloaded from the GEO database under the accession number GSE102286 and GSE101929, respectively.

## Abbreviations

NSCLC: Non-small cell lung cancer
miRNAs: microRNAs
3′-UTR: 3′-untranslated region
EMT: Epithelial-mesenchymal transition
DEGs: Differentially expressed genes
DEmiR: Differentially expressed miRNA
PPI: Protein-protein interaction
ceRNA: competing endogenous RNA
GEO: Gene Expression Omnibus
BH: Benjamini–Hochberg
FC: Fold change
KEGG: Kyoto Encyclopedia of Genes and Genomes
MRE: Mirna response element
PCA: Principal component analysis
MMPs: Matrix metalloproteinases
